# Comparison of Soil Quality Index Using Three Methods

**DOI:** 10.1371/journal.pone.0105981

**Published:** 2014-08-22

**Authors:** Atanu Mukherjee, Rattan Lal

**Affiliations:** Carbon Management and Sequestration Center, School of Environment and Natural Resources, The Ohio State University, Columbus, Ohio, United States of America; Agricultural Research Service, United States of America

## Abstract

Assessment of management-induced changes in soil quality is important to sustaining high crop yield. A large diversity of cultivated soils necessitate identification development of an appropriate soil quality index (SQI) based on relative soil properties and crop yield. Whereas numerous attempts have been made to estimate SQI for major soils across the World, there is no standard method established and thus, a strong need exists for developing a user-friendly and credible SQI through comparison of various available methods. Therefore, the objective of this article is to compare three widely used methods to estimate SQI using the data collected from 72 soil samples from three on-farm study sites in Ohio. Additionally, challenge lies in establishing a correlation between crop yield versus SQI calculated either depth wise or in combination of soil layers as standard methodology is not yet available and was not given much attention to date. Predominant soils of the study included one organic (Mc), and two mineral (CrB, Ko) soils. Three methods used to estimate SQI were: (i) simple additive SQI (SQI-1), (ii) weighted additive SQI (SQI-2), and (iii) statistically modeled SQI (SQI-3) based on principal component analysis (PCA). The SQI varied between treatments and soil types and ranged between 0–0.9 (1 being the maximum SQI). In general, SQIs did not significantly differ at depths under any method suggesting that soil quality did not significantly differ for different depths at the studied sites. Additionally, data indicate that SQI-3 was most strongly correlated with crop yield, the correlation coefficient ranged between 0.74–0.78. All three SQIs were significantly correlated (r = 0.92–0.97) to each other and with crop yield (r = 0.65–0.79). Separate analyses by crop variety revealed that correlation was low indicating that some key aspects of soil quality related to crop response are important requirements for estimating SQI.

## Introduction

A wide range of agricultural soils represents diversely managed arable lands while the main goal to improve soil quality, crop yield, and reduce the ecological foot print. Soil quality is defined as the soil’s capacity to function within natural or managed ecosystem boundaries and to sustain plant productivity while reducing soil degradation [1]–[4]. As soil quality is a complex functional concept and cannot be measured directly in the field or laboratory [5] but can only be inferred from soil characteristics [6], a range of soil parameters or indicators has been identified to estimate soil quality. However, soil quality is often related to the management goal and practices as well to soil characteristics. Thus, a mathematical or statistical framework was put forward in early 1990s to estimate soil quality index (SQI) [1], [3], [4]. The SQI was assessed so that the management goals are not only focused on productivity per se, which may result in soil degradation [7], but also on environmental issues. Thus, an appropriate SQI may have three component goals: environmental quality, agronomic sustainability, and socio-economic viability [8].

Estimation of SQI is a complex process and difficult task [9], especially when linked with several functional goals. Yet a considerable progress has been made towards estimating SQI across a number of soil types and management practices [8]–[14]. Most studies indexed soil quality employing only one method, with a few exceptions [8], [15], [16]. As computation of SQI is difficult, a strong need exists for developing a user-friendly and credible SQI through comparison of various available methods. Thus, the objective of this study was to compare SQIs computed by three methods which are conceptually different from each other. The study is based on the hypothesis that SQIs computed from three methods have similar relationship with crop yields. Data are scarce on validation of SQI against crop yield as most studies focused on the environmental aspects of the soil as end point variable and that SQI of various soil layers has not been computed [13], [16]–[18]. Nevertheless, there is a challenge in validating SQI against crop yield as SQI computed only from surface soils (0–10 and/or 0–20 cm) may not evaluate realistic relationship between soil quality and crop yield because root system can extend to deeper layers [19]. Thus, the other objective of this study was to compute SQI from multiple depths of the soil to examine its relationship with crop yield.

## Materials and Methods

### Soil sampling and analyses

Soil samples from the field (on-farm) were collected from Logan county, Ohio that included an organic (Martisco Variant silt loam: Mc, organic parent material, >26% organic matter, 40°25′12.4″N, 83°40′55.9″W), and a mineral soil (Crosby silt loam: CrB: *Fine, mixed, active, mesic Aeric Epiaqualfs*, sand: 23%, silt: 35%, clay: 42%, 40°24′52.4″N, 83°39′21.9″W), and from Franklin county, Ohio that included a Kokomo silty clay loam (Ko: *Fine, mixed, superactive, mesic Typic Argiaquolls*, sand: 9%, silt: 62%, clay: 29%, 40°00′41.2″N, 83°12′23.1″W) in May, 2013. These locations were farmer-owned field sites and before collecting the soils from specific sites permissions of the farmers were obtained from the land-owners or farmers. Initial permission was granted through The Ohio State University’s extension managers and thereafter communication was established directly by the primary author. Additionally, no endangered or protected species were involved in the current study sites, and thus no such permission was required from any other regulatory agencies. The properties of the soils and other management details were presented elsewhere [20], [21] and briefly presented in the discussion section. Soil samples were obtained from 0–10, 10–20, 20–40, and 40–60 cm depths under no-tillage (NT) and conventional tillage (CT) practices for Mc and CrB soil series and under NT for one year cover crop (CC) and no-cover crop (NCC) for Ko series. The organic soil (Mc) has been under practice of CT corn (*Zea mays*) for 10 years which was compared with the adjacent grassland soil (termed as NT). The mineral soil (CrB) was under NT and recent introduction (one year) of CT corn practices. Both soils received fertilizer (N:P:K as 5.5∶26∶30) with the rates of 336 (30, 142 and 164 kg ha^−1^ of N, P, and K, respectively) and 280 (25, 118 and 137 kg ha^−1^ of N, P, and K, respectively) kg ha^−1^ for corn, for Mc and CrB soils, respectively. The other site (Ko) was under NT corn and soybean (*Glycin max L.*) annual rotation. A mixture of pea (*Pisum sativum L.*) and turnip (*Brassica rapa L.*) cover crops with the seeding rate of 140 kg ha^−1^ was seeded prior to growing soybean in 2013. Roundup-ready soybean was seeded on 20 April, 2013 and harvested on 30 September, 2013. A row spacing of 38 cm was used during planting for corn and soybean. Soil core (inside diameter: 5.3 cm, height: 5.9 cm) samples were collected manually using soil sampler before planting (7 May, 2013) from four depths (0–10, 10–20, 20–40, and 40–60) for determination of bulk density (BD) by dividing dry mass of soil in the core by core volume [22] and hydrologic properties. Undisturbed soil cores were used to determine the water retention at field capacity (0.033 MPa), while sieved samples (<2-mm size) were used to determine the permanent wilting point (1.5 MPa). The potential available water capacity (AWC) of the soil was calculated as the difference in volumetric water content at 0.033 and 1.5 MPa moisture potentials. The water content of a soil layer was calculated by multiplying thickness of the layer with the volumetric water capacity. A minimum of three field penetration resistance (PR) measurements for soil’s mechanical strength were made for each depth using a CP40II cone penetrometer [23]. Bulk samples were obtained to measure the aggregate size distributions and fraction of water stable aggregates (WSA) using the wet sieving method [24]. Five sieves of diameter sizes 4.75, 2, 1, 0.5, and 0.25 mm openings were placed into a Yoder apparatus. Air-dried soils (5–8 mm, 51 g) were slowly wetted by capillarity action by adjusting the water level in the container so that the base of the top sieve just touched the water. The sieve combination was oscillated mechanically in the water at 60 oscillations per minute for 30 min. Aggregates retained in the sieves were transferred to glass beakers and the weight of each of five fractions was measured after drying at 60°C overnight. The data were used to compute WSA, mean weight diameter (MWD), and geometric mean diameter (GMD) [25]. Soil water retention at matrix potentials of 0.033 and 1.5 MPa were measured using a pressure plate apparatus [26]. The pH and electrical conductivity (EC) of soil were determined in 1∶2 soil:water ratio slurry using a Thermo-scientific Orion Star Series pH/Conductivity Meter. Concentrations of soil organic C and total N were determined using an Elemental analyzer (Vario Max, Elementar Americas, Inc., Germany) by the dry combustion method (900°C) after grinding subsamples to 0.25 mm. Total C and N stocks were calculated by multiplying the respective elemental concentrations by BD and the thickness of the soil layer [27].

### Soil quality index (SQI) calculations

All the SQI methods involved a set of 72 soil samples and a number of soil quality indicators as parameters. The 13 parameters used for developing SQIs were pH, EC, BD, WSA, GMD, MWD, PR, SOC concentration, N concentration, C-Stock, N-Stock, AWC, and soil water content. As some of these parameters were synthesized and redundant, only nine parameters were chosen omitting MWD, C and N concentrations, and water content from the SQI-1 and SQI-2 calculations to avoid redundancy. However, SQI-3 was not a primarily additive approach and redundancy of the parameters was eliminated through the processes of elimination as prescribed before. Thus, all 13 parameters were initially included in SQI-3 model and only four non-redundant soil parameters with maximum variations in the dataset were finally retained in the model as described in the following section. The average and standard deviation values of these parameters grouped by soil types are presented in Table 1. Under the proposed framework an ideal soil would have SQI value of 1 for the highest quality soil and 0 for the severely degraded soil [1]–[4].

**Table 1:** Descriptive statistics of all the soil indicators collected from four depths under three soil types used to estimate SQI.

Ko								
	0–10 cm		10–20 cm		20–40 cm		40–60 cm	
	Mean	SD	Mean	SD	Mean	SD	Mean	SD
pH	6.0	0.5	6.2	0.6	6.4	0.6	6.4	0.6
EC (µs cm^−1^)	190.6	151.1	165.6	84.2	151.7	71.8	175.9	92.2
BD (Mg m^−3^)	1.4	0.2	1.5	0.1	1.6	0.1	1.6	0.1
WSA %	73.1	14.8	79.5	12.0	73.3	22.5	84.7	5.6
GMD (mm)	1.4	0.4	1.6	0.4	1.4	0.3	1.5	0.3
MWD (mm)	2.6	1.7	3.1	1.8	2.3	1.7	3.4	1.1
PR (Mpa)	2.2	0.3	2.4	0.2	2.3	0.2	2.2	0.4
N (%)	0.2	0.0	0.2	0.0	0.2	0.0	0.2	0.0
SOC (%)	2.4	0.2	2.1	0.2	2.0	0.2	2.0	0.3
N-Stock (Mg/ha)	3.5	0.5	3.0	0.3	6.4	0.6	6.4	1.1
C-Stock (Mg/ha)	33.7	3.7	30.6	2.8	62.5	6.8	63.7	7.2
AWC (%)	38.5	4.1	36.7	2.1	35.8	6.4	39.9	5.8
Water content (cm)	3.8	0.4	3.7	0.2	7.2	1.3	7.9	1.0
**CrB**								
	**0–10 cm**		**10–20 cm**		**20–40 cm**		**40–60 cm**	
	**Mean**	**SD**	**Mean**	**SD**	**Mean**	**SD**	**Mean**	**SD**
pH	7.4	0.2	7.4	0.2	7.4	0.2	7.4	0.1
EC (µs cm^−1^)	250.3	44.6	217.8	26.8	212.3	31.8	223.5	45.0
BD (Mg m^−3^)	1.5	0.2	1.5	0.1	1.5	0.3	1.5	0.3
WSA %	82.4	4.4	76.4	9.7	76.5	9.5	76.5	17.7
GMD (mm)	1.2	0.1	1.1	0.2	1.1	0.2	1.2	0.3
MWD (mm)	2.0	0.5	1.7	0.8	1.5	0.7	2.0	1.1
PR (Mpa)	2.4	0.2	2.5	0.5	2.6	0.2	2.9	0.7
N (%)	0.2	0.0	0.2	0.0	0.2	0.0	0.2	0.0
SOC (%)	2.4	0.3	2.1	0.4	1.8	0.4	1.8	0.5
N-Stock (Mg/ha)	3.2	0.2	3.1	0.3	5.2	1.6	5.1	2.2
C-Stock (Mg/ha)	34.3	3.2	32.2	4.1	53.8	16.8	54.6	24.4
AWC (%)	17.8	9.0	10.1	6.8	10.7	8.2	9.0	3.2
Water content (cm)	1.8	0.9	1.0	0.7	2.1	1.6	1.8	0.6
**Mc**								
	**0–10 cm**		**10–20 cm**		**20–40 cm**		**40–60 cm**	
	**Mean**	**SD**	**Mean**	**SD**	**Mean**	**SD**	**Mean**	**SD**
pH	7.4	0.1	7.4	0.1	7.4	0.1	7.5	0.1
EC (µs cm^−1^)	616.3	40.1	570.0	92.8	633.2	126.4	599.2	129.2
BD (Mg m^−3^)	0.6	0.0	0.7	0.0	0.6	0.1	0.6	0.1
WSA %	91.2	0.5	91.0	2.5	89.7	2.5	82.5	8.7
GMD (mm)	2.1	0.0	2.2	0.0	2.2	0.0	2.2	0.1
MWD (mm)	5.5	0.1	5.5	0.2	5.7	0.4	5.0	0.5
PR (Mpa)	1.3	0.1	1.5	0.3	1.6	0.3	1.9	0.1
N (%)	1.0	0.1	0.9	0.1	0.9	0.1	1.0	0.1
SOC (%)	15.1	1.1	15.0	0.8	14.9	0.9	15.9	0.6
N-Stock (Mg/ha)	6.1	0.8	6.3	0.7	11.2	1.7	12.6	1.9
C-Stock (Mg/ha)	95.7	11.7	102.8	8.8	186.6	36.2	196.6	28.0
AWC (%)	248.3	151.3	84.8	42.4	48.2	13.8	36.1	22.1
Water content(cm)	24.8	15.1	8.5	4.2	9.6	2.8	7.2	4.4

Abbreviations: SD: standard deviation, EC: electrical conductivity, BD: bulk density, WSA: water stable aggregates, GMD: geometrical mean diameter, MWD: mean weight diameter, PR: penetration resistance, N: nitrogen, SOC: soil organic carbon, AWC: available water capacity.

### Simple additive SQI (SQI-1)

Simple additive SQI was estimated following the method outlined by Amacher et al. [28]. In this method, soil parameters were given threshold values based primarily on the literature review and expert opinion of the authors. The threshold levels, interpretations, and associated unitless soil index score values are listed in Table 2. The individual index values were then summed up to obtain a total SQI:
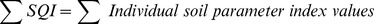
(1)


**Table 2:** Soil indicators, threshold values, interpretations and scores.

Indicators	Range	Interpretation	Score	Reference
pH	5.5–7.2	Slightly acidic to neutral: Optimum for plant growth	2	[28]
	>7.2<8.0	Slightly to moderately alkaline: Preferred by some plants,possible P and some metal deficiencies	1	
EC (us/cm)	<200	Low salt level	0	[42]
	200–500	Optimum salt level for plants	1	
	>500	High salt level, adverse effect likely	0	
BD (Mg/m^3^)	<1.0	High organic soil, supports plant roots	2	[28], [45]–[47]
	1.0–1.5	Adverse effects unlikely	1	
	>1.5	Adverse effects likely	0	
WSA (%)	<50	Infiltration and soil erosion problems likely	0	[30], [42]
	50–70	Moderate constraints	1	
	70–90	Good soil	2	
	>90	Excellent soil	3	
GMD (mm)	<1.0	Infiltration and soil erosion problems likely	0	[30], [42]
	1–2	Moderate limitations	1	
	>2	No limitation	2	
MWD (mm)	<1.0	Infiltration and soil erosion problems likely	0	[30], [42]
	1–2	Moderate limitations	1	
	2–5	Slight limitations	2	
	>5.0	No limitation	3	
PR (Mpa)	1–2	Adverse effect on plant root unlikely	2	[42], [48]
	2–3	Moderate adverse effect on plant root	1	
	>3.0	Severe adverse effect on plant roots	0	
N (%)	0.2–0.3	Moderate limitation	1	[41], [42]
	>0.3	Slight to no limitation	2	
SOC (%)	2–3	Moderate limitation	1	[41], [42]
	>3.0	Slight to no limitation	2	
N-Stock (Mg/ha)	<5.0	N deficient	1	Authors’ opinion
	5–10	Moderate to optimum N level	2	
	>10.0	N-rich soil	3	
C-Stock (Mg/ha)	<50.0	C deficient	1	Authors’ opinion
	50–100	Moderate to optimum C level	2	
	>100	C-rich soil	3	
AWC (%)	<20	Water-stress to plants	0	[31], [42]
	20–50	Moderate water availability	1	
	>50	Good water capacity for plants	2	
Water content(cm)	<5.0	Water-stress to plants	0	Authors’ opinion
	5–10	Moderate water availability	1	
	>10	Good water capacity for plants	2	

Abbreviations are same as Table 1.

The scaled SQI (SQI-1) of individual soil, was computed by Eq. 2:
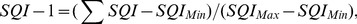
(2)whereas, SQI_Min_ = Minimum value of SQI, and SQI_Max_ = Maximum value of SQI from the total dataset.

### Weighted additive SQI (SQI-2)

In this approach, each soil parameter was first assigned unitless score ranging from o to 1 by employing linear scoring functions [8]. Non-linear scoring functions were avoided because of their lower capacity of predicting the end point variable or crop yield [8]. Soil parameters were divided into groups based on three mathematical algorithm functions: (a) ‘more is better’ (e.g., WSA, GMD, C-Stock, N-Stock, and AWC) (b) ‘less is better’ (e.g., BD, PR), and (c) ‘optimum’ (e.g., pH and EC). ‘Optimum’ properties are those which have positive influence up to a certain level beyond which the influence could be considered detrimental [13]. For ‘more is better’ parameters, each observation was divided by the highest observed value of the entire dataset so that the highest observed value would have a score of 1; for ‘less is better’ parameters, the lowest observed value in the entire dataset was divided by each observation so that the lowest observed value received a score of 1; and ‘optimum’ parameters were scored up to a threshold value as ‘more is better’, and thereafter above the threshold values were scored as ‘less is better’ [8], [29]. For example, pHs up to 5.5–7.2 were scored as ‘more is better’, and pHs >7.2 were scored as ‘less is better’.

After normalizing soil parameters, the scores were integrated into a single index value for each soil using a weighted additive approach initially suggested by Karlen and Stott [4], but modified later by Fernandes et al. [13]. The following weighted additive function was used for development of SQI-2 (Eq. 3):
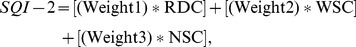
(3)where, RDC (root development capacity) is the rating for the soil’s ability to allow plant root development, WSC (water storage capacity) is the rating for the soil’s ability to store water, NSC (nutrient supply capacity) is the rating for the soil’s ability to supply nutrients. Weight 1, 2 and 3 are the respective numerical weights for each soil function (RDC, WSC, and NSC). The numerical weights were assigned to each soil function according to their importance in fulfilling the management goal(s) of maintaining soil quality. While the summation of all these numerical weights (Weight 1, 2 and 3) are supposed to be 1 and distributed evenly as 0.33, 0.33, and 0.34 [4], however, a modified approach was taken for the current study following Fernandes et al. [13], where lower weightage was given to the functional attribute which had lower number of representative indicators in the model. Similar to Fernandes et al. [13] weight values were arbitrarily chosen for RDC, WSC, and NSC as 0.4, 0.2, and 0.4 in the current study. The WSC received lower weight value than others because of low number of representative indicators (only AWC) [13] among all included parameters.

The soil parameters or indicators selected for (i) RDC were BD, PR, WSA, and GMD, (ii) WSC was AWC and (iii) NSC were pH, EC, C-Stock, and N-Stock. Within this network, the sub-weight values were given to each indicator based on their importance under the particular soil functional property, field versus laboratory measurements and scope of redundancy. The sub-weight values of different soil indicators or parameters were summed up to 1 under each soil functional property [13]. The rank of the subweight values were as follows: field measurement indicators (BD, PR) > laboratory measurement indicators (WSA, AWC, pH, EC) >> synthesized parameters (GMD, C-Stock, and N-Stock). Field indicators were given the maximum weightage as these were the most representative of the soil’s natural conditions and synthesized parameters received the lowest weightage to avoid data redundancy in the model. Thereafter, scaled SQI (SQI-2) of individual soil was computed by Eq. 2. An example of this model of development of SQI is shown in Table 3.

**Table 3:** Model of SQI-2.

Soil function	Weight	Soil Indicators	Sub-weight	Scaled score	B×C	∑ B×C	D×A	%	SQI
	A		B	C		D			
RDC	0.4	BD	0.35	0.41	0.14	0.46	0.18	52.94	0.34
		PR	0.35	0.52	0.18				
		WSA	0.20	0.54	0.11				
		GMD	0.10	0.34	0.03				
WSC	0.2	AWC	1.00	0.06	0.06	0.06	0.01	2.94	
NSC	0.4	pH	0.30	0.79	0.24	0.38	0.15	44.12	
		EC	0.30	0.25	0.08				
		C-stock	0.20	0.11	0.02				
		N-stock	0.20	0.18	0.04				

Abbreviations: RDC: root development capacity, WSC: water storage capacity, NSC: nutrient storage capacity; all other abbreviations are same as Table 1.

### Statistically modeled SQI (SQI-3)

A statistics-based model was used to estimate SQI using principal component analysis (PCA) [8], [12], [14], [18], [30], [31]. The PCA-model is used to create a minimum data set (MDS) to reduce the indicator load in the model and avoid data redundancy [8]. The main difference between the first two versus the PCA method is that the first two rely mainly on subjective expert opinion and literature review, while the PCA method is more objective of using a number of statistical tools (multiple correlation, factor and cluster analyses) which could avoid any biasness and data redundancy by choosing an MDS using mathematical formulae [8], [32]. A relative advantage and disadvantage of each SQI method is explained in the later section. The preliminary function of PCA is to reduce the dimensionality of the entire data set consisting of a large number of interrelated variables, while retaining as much as possible of the variations present in the data set. This is achieved by transformation to a new set of variables, the principal components (PCs), which are uncorrelated, and ordered so that the first few retain most of the variation present in all of the original variables [33], [34]. In other words, the PCA method was chosen as a data reduction tool to select the most appropriate indicator(s) to represent and estimate SQI [18].

**Figure 1:**
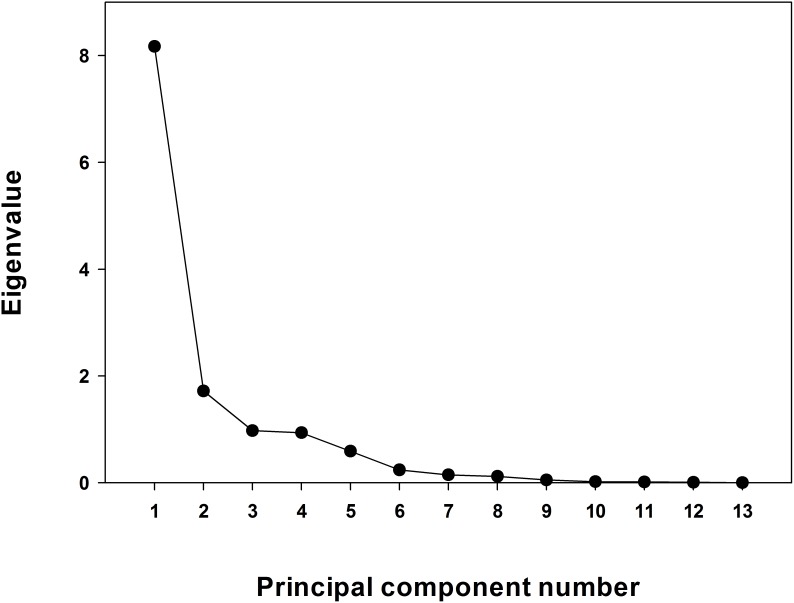
Scree plot of principal component analyses.

All the original observations (untransformed data) of each soil were included in the PCA model using SPSS, version 21.0 [35]. The PCs with high eigenvalues represented the maximum variation in the dataset [8], [12], [14], [18], [30], [31]. While most studies have assumed to examine PCs with eigenvalue >1.0 following Kaiser [36], the present study had the third and fourth components with eigenvalues of 0.98 and 0.89 and variances >5% (Table 4, Fig. 1). These were examined with first two PCs (eigenvalues 8.23, and 1.67) as prescribed for the cases of fewer than three components with eigenvalue >1.0 [8], [37]. Under a given PC, each variable had corresponding eigenvector weight value or factor loading (Table 2). Only the ‘highly weighted’ variables were retained to include in the MDS (Table 2). The ‘highly weighted’ variables were defined as the highest weighted variable under a certain PC and absolute factor loading value within 10% of the highest values under the same PC [37]. Thus, the bold-face values (Table 4: EC, BD, GMD, MWD, SOC, and N for PC-1, AWC for PC-2, WSA for PC-3, and pH for PC-4) were considered highly weighted eigenvectors and therefore were initially selected in the MDS. However, when more than one variable was retained under a particular PC, multivariate correlation matrix (Table 5) was used to determine the correlation coefficients between the parameters [8], [12]. If the parameters were significantly correlated (r>0.60, *p*<0.05), then the one with the highest loading factor was retained in the MDS and all others were eliminated from the MDS to avoid redundancy. Following this procedure, except SOC, all other eigenvectors from PC-1 were eliminated from MDS due to high and significant correlation between each other (Table 5). The non-correlated parameters under a particular PC were considered important and retained in the MDS [10], [12]. All the bold-faced and underlined soil parameters in Table 4 were selected in the final MDS.

**Table 4:** Results of principal component analyses (PCA).

	PC-1	PC-2	PC-3	PC-4
Eigenvalues	8.23	1.67	0.98	0.89
% Variance	63.34	12.87	7.56	6.82
Cumulative variance	63.3	76.2	83.8	90.6
**Eigen vectors or factor loading**				
pH	0.423	0.351	–0.378	**0.711**
EC	**0.925**	0.113	–0.153	0.147
BD	**–0.881**	–0.034	0.212	0.002
WSA	0.590	–0.020	**0.669**	0.343
GMD	**0.927**	0.024	0.276	–0.024
MWD	**0.904**	0.001	0.342	0.046
PR	–0.770	0.268	–0.131	0.092
SOC	**0.975**	0.073	–0.109	–0.025
N	**0.972**	0.050	–0.103	–0.053
C-Stock	0.831	0.412	–0.107	–0.268
N-Stock	0.722	0.440	–0.067	–0.385
AWC	0.555	**–0.782**	–0.189	0.057
Water content	0.624	–0.693	–0.199	–0.066

Abbreviations are same as Table 1; PC: principal component; bold values under each component are highly weighted and underlined bold values are selected in minimum data set.

**Table 5:** Pearson correlation coefficients (r) for all soil indicators.

Correlation (r) matrix	pH	EC	BD	WSA	GMD	MWD	PR	N	SOC	N-Stock	C-Stock	AWC
EC	0.547											
BD	−0.394	−0.88										
WSA	0.248	0.483	−0.338									
GMD	0.279	0.8	−0.762	0.673								
MWD	0.286	0.782	−0.725	0.734	0.975							
PR	−0.129	−0.655	0.684	−0.456	−0.71	−0.687						
N	0.405	0.922	−0.908	0.47	0.877	0.835	−0.737					
SOC	0.438	0.93	−0.914	0.473	0.881	0.842	−0.731	0.995				
N-Stock	0.271	0.632	−0.551	0.32	0.642	0.595	−0.396	0.709	0.707			
C-Stock	0.382	0.764	−0.702	0.367	0.742	0.695	−0.489	0.822	0.833	0.965		
AWC	0.091	0.447	−0.449	0.275	0.439	0.441	−0.525	0.504	0.489	0.106	0.181	
Water content (cm)	0.092	0.487	−0.485	0.282	0.501	0.496	−0.557	0.558	0.546	0.28	0.333	0.967
**Significance level**												
EC	<0.001											
BD	<0.001	<0.001										
WSA	0.018	<0.001	0.002									
GMD	0.009	<0.001	<0.001	<0.001								
MWD	0.007	<0.001	<0.001	<0.001	<0.001							
PR	0.141	<0.001	<0.001	<0.001	<0.001	<0.001						
N	<0.001	<0.001	<0.001	<0.001	<0.001	<0.001	<0.001					
SOC	<0.001	<0.001	<0.001	<0.001	<0.001	<0.001	<0.001	<0.001				
N-Stock	0.011	<0.001	<0.001	0.004	<0.001	<0.001	<0.001	<0.001	<0.001			
C-Stock	<0.001	<0.001	<0.001	0.001	<0.001	<0.001	<0.001	<0.001	<0.001	<0.001		
AWC	0.224	<0.001	<0.001	0.011	<0.001	<0.001	<0.001	<0.001	<0.001	0.195	0.07	
Water content (cm)	0.22	<0.001	<0.001	0.009	<0.001	<0.001	<0.001	<0.001	<0.001	0.01	0.003	<0.001

Abbreviations are same as Table 1.

After selection of parameters for the MDS, all selected observations were transformed using linear scoring functions (less is better, more is better and optimum) as described in SQI-2 method. Once the selected observations were transformed in numerical scores (ranged 0–1), a weighted additive approach was used to integrate them into indices for each soil [8], [12]. Each PC explained a certain amount of variation in the dataset (Table 4), which was divided by the maximum total variation of the all PCs selected for the MDS to get a certain weightage value under a particular PC [8]. For example, the % variance (63.3) was divided by total cumulative variance (90.6) to obtain the weight value of 0.7 for PC-1 (Table 4). Thereafter, the weighted additive SQI was computed using Eq. 4:
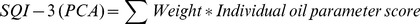
(4)


### Validation of SQI

The SQIs estimated from three different methods were validated against soybean (*Glycin max*) and corn (*Zea mays*) yield data of that particular year of collection of soil and crops by computing correlation coefficients [8]. The SQI values were also compared through pearson correlation to understand the effectiveness of each other.

### Statistical analyses

All values are presented as means ± standard deviations of three field or laboratory measurements. Significant differences between treatments were analyzed using Tukey’s test in PROC GLM in SAS version 9.2 [38]. Treatment differences were deemed significant at *p*<0.05. The PCA was performed in SPSS version 21 [35]. Descriptive statistics and linear regressions were computed in Microsoft Excel [39] and all the figures were obtained using Sigmaplot Version 12.0 [40].

## Results and Discussions

Most data of three farm sites have been presented and the detailed discussions of soil type, crop and management effects on soil properties are discussed elsewhere [20], [21]. The mean and standard deviation values of the soil parameters by depth under three soil types are listed in Table 1. Most properties of mineral soils (Ko and CrB) were significantly different compared to those of the organic soil (Mc) and except for AWC and water content two mineral soils’ characteristics were similar across soil profile up to 60 cm depth (Table 1). The water characteristics of Ko soil were significantly higher than those of CrB. On the other hand, organic soil (Mc) had much improved (lower BD and PR, and higher WSA, SOC, N concentrations and stock, AWC and water content) soil properties compared to those of the mineral counterparts in all four different depths (Ko and CrB). Impacts of all these parameters on overall soil quality in four soil depths calculated by three indexing methods are discussed in the following sections.

**Figure 2:**
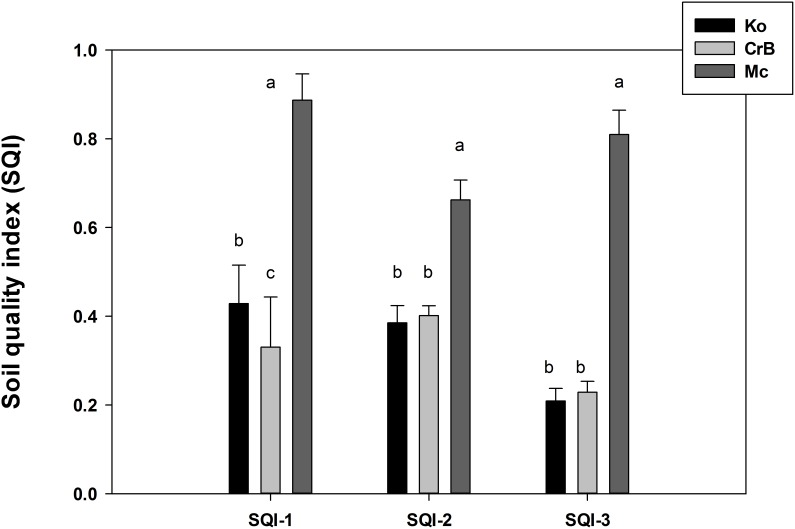
Overall soil quality index (SQI) values under different soil types. Different letters indicate significant differences at p<0.05 level for particular indexing method.

Under the framework of SQI-1, nine soil quality indicators were integrated numerically after scoring them primarily using the information from literature review. However, scoring data on some physicochemical indicators (e.g., C and N stocks) is scarce in the literature and thus, authors’ opinion was used in those cases. Further, different studies used different scoring on the same indicator based on soil type, and management goals. For an example, while Amacher and Perry [28] had eight different range classes and scores for pH, Feiza et al. [41] had only four classes and scores available for pH. Thus, scoring on indicators in the current study (Table 2) had importance on experts’ opinion although the knowledge was primarily based on available literature and authors’ experience [28], [42]. The high variability in the observations (Table 1) was reflected in the SQI-1 values. There was no statistical difference (*p*>0.05) across treatments (NCC versus CC, and CT versus NT) and depth under specific soil type (Table 6). Overall performance of indexing methods was determined from averaging SQI values obtained from calculations for each depth and presented in Fig. 2. The, SQI-1 was significantly higher in the following order: Mc>Ko>CrB (Fig. 2), indicating that soil quality was influenced more by soil type than management and sampling depth under the SQI-1 approach.

**Table 6:** Treatment effects on soil quality index (SQI) under three soil types (Ko: Kokomo, CrB: Crosby silty loam, Mc: Muck) and four soil depths.

Treatments									
Ko		NCC	CC	NCC	CC	NCC	CC	NCC	CC
		0–10 cm		10–20 cm		20–40 cm		40–60 cm	
	SQI-1	0.36a	0.36a	0.40a	0.40a	0.45a	0.48a	0.48a	0.50a
	SQI-2	0.40a	0.37a	0.39a	0.36a	0.40a	0.36a	0.43a	0.38a
	SQI-3	0.21a	0.23a	0.20a	0.22a	0.19a	0.20a	0.21a	0.21a
**CrB**		**CT**	**NT**	**CT**	**NT**	**CT**	**NT**	**CT**	**NT**
		**0–10 cm**		**10–20 cm**		**20–40 cm**		**40–60 cm**	
	SQI-1	0.36a	0.38a	0.36a	0.17a	0.45a	0.29a	0.38a	0.26a
	SQI-2	0.42a	0.40b	0.40a	0.37a	0.41a	0.40a	0.41a	0.40a
	SQI-3	0.26a	0.24a	0.24a	0.22a	0.22a	0.22a	0.22a	0.21a
**Mc**		**CT**	**NT**	**CT**	**NT**	**CT**	**NT**	**CT**	**NT**
		**0–10 cm**		**10–20 cm**		**20–40 cm**		**40–60 cm**	
	SQI-1	0.88a	0.88a	0.90a	0.90a	0.90a	0.90a	0.90a	0.81a
	SQI-2	0.70a	0.72a	0.65a	0.61a	0.65a	0.66a	0.67a	0.65a
	SQI-3	0.85a	0.85a	0.82a	0.78a	0.78a	0.77a	0.82a	0.81a

Abbreviations: NCC: no cover crop, CC: cover crop, CT: conventional tillage, NT: no tillage.

Different letters under specific row and depth indicate significant differences at *p*<0.05 level, underline value is significant at *p*<0.1 level.

**Figure 3:**
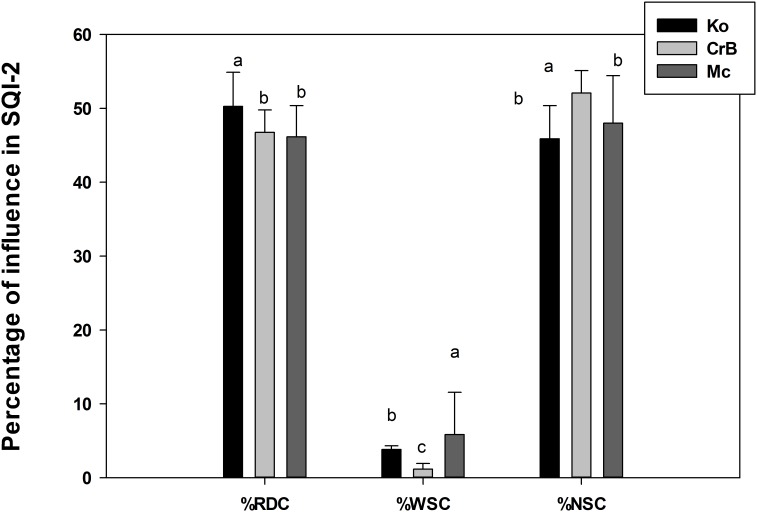
Percentage contribution of each soil function in SQI-2 under different soils. Different letters indicate significant differences at p<0.05 level for particular soil function. Abbreviations: RDC: root development capacity, WSC: water storage capacity, NSC: nutrient storage capacity.

**Figure 4:**
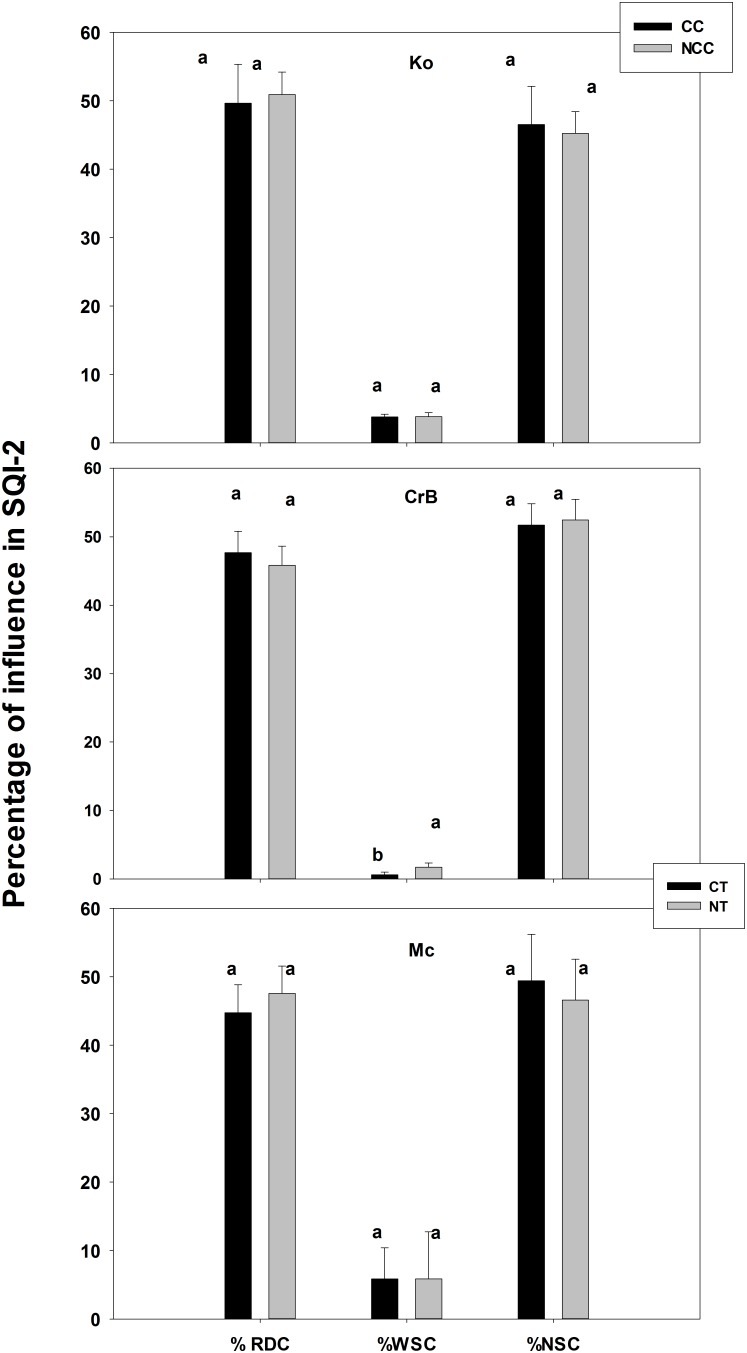
Percentage contribution of each soil function in SQI-2 under various management practices in different soil. Different letters indicate significant differences at p<0.05 level for particular soil function. Abbreviations: RDC: root development capacity, WSC: water storage capacity, NSC: nutrient storage capacity; NCC: no cover crop, CC: cover crop, CT: conventional tillage, NT: no tillage.

Overall percentage of various soil functional influences in SQI-2 is presented in Fig. 3 and depthwise percentage of influence in SQI-2 is presented in Fig. S1. Generally, mineral soils (Ko and CrB) did not have any depthwise significant changes for WSC but 0–10 cm layer of organic (Mc) soil had significantly higher influence of WSC in SQI-2 compared to lower depths. On the other hand, while both Ko and Mc had significantly higher influence of RDC in SQI-2 in the upper layers than the lower depths, the opposite trend was found for the NSC (Fig. S1). In the general composition of SQI-2, the main contributors were RDC, which ranged from 39–57% and NSC ranged from 35–59%. However, the WSC was relatively small and ranged merely from 0.1–12% (Fig. 3). The relative influence of root development functions in SQI-2 has been significantly higher in Ko than CrB, and Mc by 8 and 9%, respectively and the same for nutrient storage functions in SQI-2 has been significantly higher in CrB than Ko and Mc by 13 and 8%, respectively (Fig. 3). On the other hand, Mc soil had significantly higher water storage functional contribution to SQI-2 by 53 and 500%, respectively, compared to that of mineral (Ko and CrB) soils (Figs. 3). Thus, while the mineral soil quality was more sensitive to the functional attributes dedicated to root development and nutrient storage, muck soil quality was more influenced by soil attributes related to water storage under the scheme of SQI-2. Further, there was no significant effect on management practices on %RDC and %NSC to SQI-2 in different soil/management/crop combinations (Fig. 4). Note that CC/NCC management was used on field cultivated to soybean but CT/NT was used to soil under corn. However, contribution of WSC to SQI-2 for the case of CrB soil under NT management was significantly higher (178%) than that of CT practice (Fig. 4).

Correlation coefficients of SQIs to each other and depthwise SQIs and dry grain and straw are given in Table 7. Crop yield is obviously related to soil quality but soil quality in different layers may not be same and can influence crop yield accordingly. Data on SQI variation for different soil layers are scarce and thus no comparison to the data presented in the current study (Table 6) was possible. However, while numerical values of SQI were almost unchangeable for different depths at Mc site, SQI generally variable with depth in Ko and CrB sites (Table 6). One goal of this study was to validate SQI against crop yield as only a few studies have done so with some exceptions [8], [14]. Challenge lies in establishing a correlation between crop yield versus SQI calculated either depthwise or in combination of soil layers as standard methodology is not yet available and was not given much attention to date. For example, one can have only one crop yield value for multiple soil layers and thus to date crop yield has been correlated only against SQI calculated from surface layer. Low (ranged from 0.11–0.32) and high (ranged from 0.83–0.96) correlation coefficients of SQI and crop yield across a number of soil/crop combinations were reported [8], [31]. The reasons attributed to low correlation between yield and SQI were: (i) the assumptions that the indicators considered good for a soil quality may not always lead to desired outcomes and flexibility in scoring functions needed [8], (ii) indicators which may not directly relate to crop performance were given more weightage by the method employed, or (iii) some key soil indicators were not included in the MDS due to the design of the study [9], [43]. Nevertheless, in order to evaluate the index performance in the current study, SQIs were correlated against end point variable (i.e., yield) at variable depths (Table 7). In the present study, depth of soil sampling had no significant effect in SQI values (Table 6) and thus the individual SQI values were integrated by combining 0–20 cm and 0–60 cm profiles by averaging 0–10, and 10–20 cm for obtaining values for 0–20 cm depth and 0–10, 10–20, 20–40 and 40–60 cm for obtaining values for 0–60 cm depths and correlated the combined values with crop yield (Table 7). In the present study, grain and biomass yields were all significantly (*p*<0.05, r = 0.65–0.79) correlated under any SQI methods employed indicating that either of these methods was successful in predicting crop yield (Table 7). However, correlation coefficients in Table 7 are derived from the complete datasets including both corn and soybean yield but the correlation was considerably low (r<0.20 for soybean grain and r<0.60 for corn grain; data not shown), when dataset was further divided into corn and soybean. As discussed above, low correlation of SQI and crop yield was observed before and one of the listed reasons was some key soil indicators were not included in the dataset as per the experimental design and objective [8]. Crop yield is invariably related to soil fertility status and thus SQI may not always lead to high correlation with yield if some of the related characterizations are not observed as identified before [9], [43] which is supported by the data of the current study (Table 7). Thus, the low correlation coefficient values may be because soil fertility (ion exchange capacities, macro and micro nutrients) and microbial (microbial biomass, microbial C, and N, and soil respiration) aspects were not monitored in the current studied sites, especially in the case of soybean grain yield. Nevertheless, relatively higher correlation coefficient was observed in the 0–10 cm soil layer than the deeper soil profile (Table 7) probably due to higher plant root density in the top layer of the soil [44].

**Table 7:** Pearson correlation coefficients (r) of soil quality index (SQI) versus crop yield and correlations between different SQI values which were averaged up to a certain depth; all numbers are significant at *p*<0.05 level.

SQI-1	Dry Grain	Dry Straw
0–60 cm	0.65	0.67
0–20 cm	0.71	0.71
0–10 cm	0.73	0.71
**SQI-2**	**Dry Grain**	**Dry Straw**
0–60 cm	0.75	0.73
0–20 cm	0.75	0.72
0–10 cm	0.79	0.75
**SQI-3**	**Dry Grain**	**Dry Straw**
0–60 cm	0.76	0.74
0–20 cm	0.76	0.74
0–10 cm	0.78	0.76
	**SQI-1**	**SQI-2**
SQI-2	0.92	
SQI-3	0.93	0.97

Additionally, SQI computed from different methods were also highly correlated with each other (Table 7) suggesting that (i) a relatively easy and user-friendly SQI (SQI-1) can be computed to evaluate and compare soil quality, which is similarly useful to other approaches, (ii) giving certain appropriate weightage on scores (SQI-2) could be similarly useful in predicting particular soil quality and (ii) a minimum group of carefully selected soil indicators (SQI-3) may be used to evaluate soil quality across various soil types and management practices. As all three SQI methods were significantly correlated to each other, it is difficult to conclude which one is the best approach, however, it highly depends on factors such as design of the study, choice of soil parameters included in the model to compute SQI and end point variable (environmental aspects versus crop yield). The advantage of using SQI-1 is that the soil quality could be assessed after measuring any number (low to high) of soil parameters and this procedure is relatively easier compared to other methods as the scoring requires literature review and expert opinions only. The disadvantage of SQI-1 is that it is subjective and relies mainly on researcher’s point of view. On the other hand, advantage of SQI-2 is that it includes weightage based on the design of the study, system or the dataset to offset the subjectivity of the approach present in SQI-2. However, the disadvantage of SQI-2 is that it requires multiple numbers of soil parameters under different soil functional systems which may be expensive and time consuming in practical case. The SQI-3 is advantageous in the aspect of its ability to predict soil quality based on a reduced dataset with low number of soil parameters. Additionally, it is mostly objective approach as the statistical procedure would select a low number of soil parameters needed to calculate SQI based on the variances present in the whole dataset. So, in a long-term aspect within a particular soil/crop system, SQI-3 can be used effectively once it evaluated the most influential soil parameters required to assess soil quality of a particular soil/crop/management scenario. However, current data suggest that relative higher SQI values were obtained for Ko and Mc by SQI-1 than SQI-2 or SQI-3 and for CrB by SQI-2 than SQI-1 or SQI-3 (Fig. 2). Although SQI-1 and SQI-2 had higher values than SQI-3 in different scenarios, however, based on the current experimental design, and selected soil parameters SQI-3 successfully predicted crop yield relatively higher [Table 7 and also when grouped by crops (data not shown)] than SQI-1 and 2. Depth had little influence in any SQIs in the present study and additional chemical and biological parameters are also needed to strengthen the validity of SQI. Thus, SQI-3 appears to be the best method among the three under long-term scenario, especially due to its objective approach, relative higher correlations with crop yield and lower number of indicator selection which is more cost and time-effective over time than other approaches.

## Conclusions

The data presented support the following conclusions: The SQI calculated by three different methods indicated that studied muck soil has significantly higher soil quality than that of mineral soils under on-farm conditions. The SQI was affected more by management and soil type than by depths in the studied on-farm sites. The SQI computed using three established methods were all significantly correlated to each other indicating that relatively easy and user-friendly SQI (SQI-1) can be similarly useful to evaluate soil quality, appropriate weightage on scores can predict soil’s quality (SQI-2) with high performance but requires a number of soil parameters under different soil functional components and carefully chosen MDS with small numbers of soil variables (SQI-3) may adequately evaluate its quality. All three SQIs were highly and significantly correlated with crop yield. In general, however, analyses by crop type (corn and soybean) revealed that correlation was low (r<0.20 or 0.60 for soybean or corn grain yield, respectively; data not shown) suggesting choice of crop-specific and key soil parameters used in computing SQI. Under the current experimental on-farm conditions SQI-1 (for Ko and Mc) and SQI-2 (for CrB) values were higher than SQI-3, however, SQI-3 can be regarded as the best and easiest model given its relatively higher success to predict crop yield and objectivity approach with lower number of indicator selection ability which should be regarded as a relatively less expensive procedure over time compared to SQI-1 and 2. In addition, in order to effectively predict particular crop yield one must include soil fertility and microbial parameters in the model of SQI.

## Supporting Information

Figure S1 **Percentage contribution of each soil function in SQI-2 under different soils in four soil layers; different letters indicate significant differences at p<0.05 level for particular soil function.** Abbreviations: RDC: root development capacity, WSC: water storage capacity, NSC: nutrient storage capacity.
(TIF)
