# Supplementary figures and images for: Comparison of Soil Quality Index Using Three Methods
Source: PLoS One. 2014 Aug 22;9(8):e105981. doi: 10.1371/journal.pone.0105981 (PMC4141864; doi:10.1371/journal.pone.0105981)

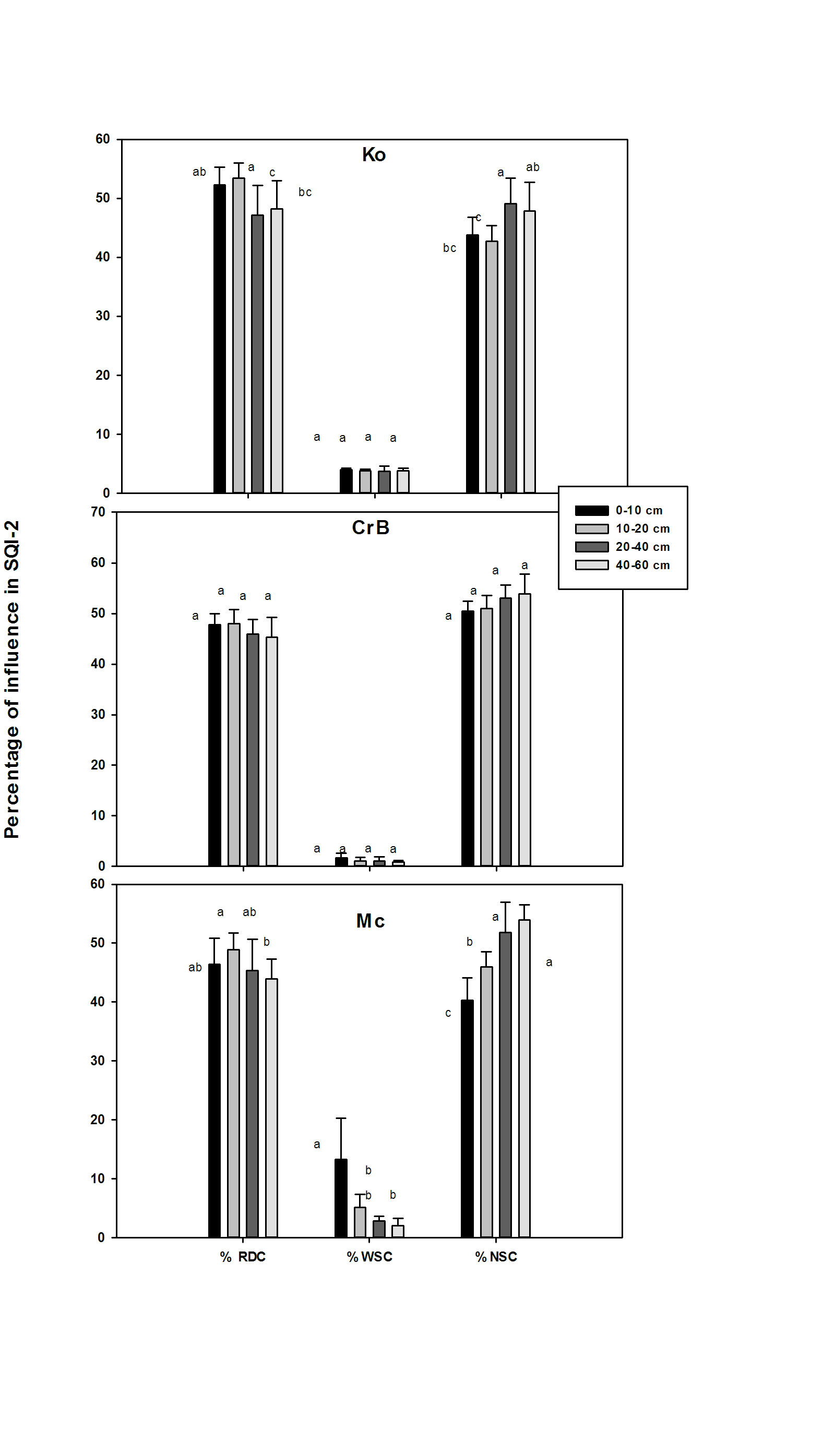

Supplement: Figure S1 — Percentage contribution of each soil function in SQI-2 under different soils in four soil layers; different letters indicate significant differences at p<0.05 level for particular soil function. Abbreviations: RDC: root development capacity, WSC: water storage capacity, NSC: nutrient storage capacity. (TIF) [file pone.0105981.s001.tif]
